# Psychometric Evaluation of the Swedish Version of the Prolonged Grief Disorder-13 (PG-13) in a Bereaved Mixed Trauma Sample

**DOI:** 10.3389/fpsyt.2020.541789

**Published:** 2020-12-03

**Authors:** Josefin Sveen, Kristina Bondjers, Julia Heinsoo, Filip K. Arnberg

**Affiliations:** ^1^Department of Neuroscience, National Centre for Disaster Psychiatry, Psychiatry, Uppsala University, Uppsala, Sweden; ^2^Department of Health Care Sciences, Palliative Research Centre, Ersta Sköndal Bräcke University, Stockholm, Sweden; ^3^Stress Research Institute, Stockholm University, Stockholm, Sweden

**Keywords:** prolonged grief, PG-13, loss, trauma, factor analysis, latent structure

## Abstract

**Background:** This study aimed to examine the psychometric properties of the Swedish PG-13 in a bereaved trauma exposed sample. A second aim was to examine the latent structure of prolonged grief using the PG-13.

**Methods:** The participants were adults (*n* = 123) taking part in an ongoing longitudinal study regarding the effects of potentially traumatic events. Participants had experienced a potentially traumatic event in the past 5 years and had reported a death of a significant other either as their primary traumatic event or in addition to another traumatic event. Assessment included self-report of prolonged grief, posttraumatic stress, and general psychological distress. Clinical interviews were used to assess depression, posttraumatic stress disorder, and disability level. The psychometric properties of the Swedish PG-13 were examined through reliability tests and assessment of associations with symptoms of posttraumatic stress, depression, general psychological distress, and disability level. Principal component analysis (PCA) and confirmatory factor analyses (CFA) were used to assess the latent structure.

**Results:** The internal consistency (Cronbach's α = 0.86) and test-retest (*r* = 0.86) reliability were good. PCA suggested a three-factor model as descriptive of the latent structure of the instrument. Therefore, the CFA used this model, as well as two models suggested in the literature. The three-factor model had the best fit to data. Support of concurrent validity of PG-13 was shown by moderate positive associations with measures of posttraumatic stress, depression, and general psychological distress.

**Conclusions:** The Swedish PG-13 demonstrated good psychometric properties, and its use in research and practice to assess prolonged grief was supported. The factor analyses provided stronger support for models with two or three factors, as compared with a unidimensional model of prolonged grief, with the three-factor model having the best fit.

## Introduction

A significant minority of bereaved individuals experience persistent and severe symptoms of grief that do not diminish with time (1, 2). Chronically disabling and distressing grief reactions have, under various names, attracted research interest—most recently as Prolonged Grief Disorder (PGD). Prigerson et al. (2) proposed diagnostic criteria for PGD to be included in the 11th edition of Internal Classification of Diseases (ICD-11) and 5th version of Diagnostic and Statistical Manual of Mental Disorders [DSM-5; (3)]. However, in the DSM-5, a disorder called persistent complex bereavement disorder was included for further study (PCBD; 3), with symptoms divided into core symptoms (e.g., yearning, sorrow, emotional pain), reactive distress to the death (e.g., difficulty accepting the loss, bitterness related to the loss, avoidance of reminders of the loss), and social/identity disruption (e.g., difficulty trusting others, feeling life is empty without the deceased, confusion about one's role in life). The more recently released ICD-11 (4) includes Prolonged Grief Disorder as a disorder specifically associated with stress, differing from the PGD proposed by Prigerson et al. (2). PGD in ICD-11 is characterized by persistent and intense longing for the deceased, preoccupation with the deceased, additional symptoms suggestive of emotional pain, and a difficulty to accept the loss (5). These responses should be associated with functional impairment and persist for at least 6 months after the loss (6).

A meta-analysis by Lundorff et al. (7) found that one out of 10 bereaved adults, following a non-violent loss, is at risk of developing PGD, whereas a recent meta-analysis by Djelantik et al. (8) showed that almost half of the bereaved adults experienced PGD following unnatural losses. Previous research suggests that while PGD shares features with both depression and post-traumatic stress disorder (PTSD), it also provides unique explanatory power (2, 9–11) and is independently related to decreased mental and physical health (12–16). However, uncertainty remains regarding PGD in people with a history of traumatic events. For example, it is uncertain if the qualities of PGD mentioned above are found also among bereaved individuals who report a traumatic index event not related to bereavement.

Prigerson et al. (2) created a diagnostic self-rating instrument for their proposed criteria for PGD, the Prolonged Grief questionnaire-13 (PG-13). However, there are few studies examining the psychometric properties of the PG-13. One study has shown that the PG-13 has good psychometric properties with high sensitivity and specificity (93.3 and 98.3%) for bereaved adults (17). The Swedish PG-13 has shown satisfactory psychometric properties in a sample of cancer-bereaved parents, with the latent structure of PG-13 being explained by a unidimensional structure (18). In this study, the Swedish PG-13 was slightly adopted to bereaved parents by changing the wording “the person you lost” to “the child you lost.”

There are several studies examining the latent structure of prolonged grief using different measures, such as the Inventory of Complicated Grief and the revised version of Inventory of Complicated Grief. Most studies have found support for a unidimensional structure of prolonged grief (2, 9, 10, 19, 20). In a sample of older adults, O'Connor et al. (21) examined the one-factor model, as well as a theoretically derived two-factor model (i.e., separation distress and traumatic distress), and found that the two-factor model had a better fit. However, the two factors were highly correlated and the one-factor model had an acceptable fit. Newson et al. (22) found four factors in a sample of older adults.

As the psychometric properties of the Swedish PG-13 has only been examined with a slightly modified version in bereaved parents, additional validation should be conducted with more heterogenous populations. This study aimed to examine the psychometrics of the Swedish PG-13 in trauma exposed sample, who had also experienced bereavement. Internal consistency and test-retest reliability were evaluated, as was the concurrent validity of the PG-13 in relation to posttraumatic stress disorder, depression, and general psychological distress. A second aim was to examine the latent structure of prolonged grief using the PG-13.

## Materials and Methods

### Participants and Procedure

The participants in the current study were individuals taking part in an ongoing longitudinal study regarding the effects of potentially traumatic events—the Traumatic Events in a Longitudinal Survey (TRACES) study—at Uppsala University, Sweden. TRACES aims to examine psychological reactions to adverse events among health care and non-health care seeking individuals and how such reactions fluctuate over time.

Participants were self-recruited from the general public in two urban counties in Sweden via advertisements in local and social media, flyers at primary care and outpatient psychiatric care, as well as via information from health-care providers. Participants signed up for the study via health care staff, who provided the research group with contact information, or via an online form. Inclusion criteria for the longitudinal study were: being ≥18 years of age, having experienced a potentially traumatic event in the last 5 years, and being able to understand and speak Swedish. Exclusion criteria were ongoing traumatic event or stressors (e.g., ongoing violence, disease), and concurrent severe mental illness (e.g., ongoing psychosis). Participants were compensated with two movie tickets. For the current study, participants who reported bereavement of a significant other (i.e., a family member, close relative, or close friend) were drawn from the longitudinal study. No criteria were issued on the relationship to the deceased.

All participants were screened via telephone with questions about the nature of their potentially traumatic events and additional questions related to the inclusion and exclusion criteria. Participants then responded to a survey, including several questionnaires, either via a secure online web portal or on paper. After the survey was filled out, the participants took part in structured face-to-face interviews conducted by licensed psychologists, psychologists in training, or master's students in clinical psychology under the supervision of licensed psychologists. All interviewers were trained in the interview protocols by the researchers. The interview sessions encompassed assessment of the participants' history of potentially traumatic events, and structured clinical interviews, as detailed below. During the interview, participants who reported having experienced a loss of a significant other filled in the Swedish version of PG-13. After their interview, each participant was given a retest questionnaire to complete the following day, at the earliest, and return by post.

### Measurements

#### PG-13

The PG-13 is comprised of 13 items which follow the diagnostic criteria suggested by Prigerson et al. (2). The questionnaire assesses symptoms of separation distress and symptoms of cognitive, behavioral, and emotional changes, as well as the duration of these symptoms and perceived functional impairment. The first four items concern symptoms—yearning, intense grief, avoidance, and numbness—during the preceding month, rated on a five-point frequency scale (*Not at all* to *Several times a day*), with the fifth item establishing whether yearning or intense grief have been present at least daily beyond 6 months after the loss (*No*/*Yes*). The next seven items concern confusion with one's role in life, difficulties accepting the loss, loss of trust, bitterness, difficulties moving on in life, emotional isolation, and meaninglessness. These items are rated on a five-point intensity scale (*Not at all* to *Overwhelmingly*). Lastly, one item asks about deterioration in everyday function (*No*/*Yes*). A total symptom severity score is computed by giving the 11 symptom items (items 1, 2, 4–12) a score of 1 to 5 and summing up these scores. Thus, symptom severity ranges from 11 to 55. A dichotomous scoring for PGD screening is also used. For a positive screening, the respondent must endorse yearning or intense grief at least once a day (items 1–2), at least 6 months after the loss (item 3), experience at least five of the remaining symptoms at least “daily” (items 4–5) or “a lot” (items 6–12), and report functional impairment (item 13). The PG-13 has previously been translated into Swedish at the National Center of Disaster Psychiatry at Uppsala University. The original PG-13 was translated into Swedish by a professional translator and then revised by lic. clin. psychologists with extensive experience of grief. A second professional translator, a native English speaker with a PhD in psychology, did a back-translation of the final Swedish version. This person was blind to the English original. The psychometric properties of the Swedish version of PG-13 has been examined in cancer-bereaved parents (18).

#### Clinician-Administered PTSD Scale for DMS-5 (CAPS-5)

The CAPS-5 (23) is a structured clinical interview considered to be the gold standard for diagnostic assessment of PTSD. The interview assesses PTSD symptoms, as described in DSM-5, within the preceding month. It contains 20 symptom items, each assessed on a five-point Likert scale from 0 (*Not present*) to 4 (*Extreme*). The total symptom severity ranges from 0 to 80. CAPS-5 also assesses functional disability due to PTSD symptoms and the duration of the disorder.

#### The PTSD Checklist for DSM-5 (PCL-5)

The PCL-5 (24) was used as a self-report measure of PTSD symptoms during the preceding month. It contains 20 items, each symptom corresponding to a symptom in DSM-5. Items ask how much respondents have been bothered by each symptom during the preceding month, and are answered on a five-point Likert scale from 0 (*Not at all*) to 4 (*Extremely*). The total symptom score ranges from 0 to 80. The Swedish version of PCL-5 has been shown to have good psychometric properties (25).

#### The Symptom Checklist 27 (SCL-27)

The SCL-27 (26) was used as a measure of general psychological distress. It is a self-report scale and measures symptoms on six subscales: depressive, dysthymic, somatization, mistrust, social phobic, and agoraphobic symptoms. Each subscale includes four to six items. All items are answered on a four-point Likert scale and the total severity score ranges from 0 to 108. In this study, both the subscales and the total score were used in the analyses of the concurrent validity of the PG-13.

#### MINI Neuropsychiatric Interview (MINI 7.0)

MINI 7.0 (27, 28) is a screening interview for several psychiatric disorders. The data from the depression module, ongoing and previous depression, were used in the current study. The number of depression criteria fulfilled was used to assess the level of depression symptoms; it ranges from 0 to 9.

#### World Health Organization Disability Assessment Scale (WHODAS 2.0)

WHODAS 2.0 is a structured interview assessing a person's disability levels in several domains. Items are scored from 0 (*No difficulties*) to 4 (*Extreme difficulty/cannot do*). The current study employed the 12+24 version, where the interview is discontinued if the interviewee responds negatively to the first six to 12 items, and the remaining items are scored 0. If a respondent reports disability in any domain, additional domain-specific items are given and scored. The complex scoring method suggested by WHO was used, which provides a total score ranging from 0 (*No disability*) to 100 (*Full disability*). WHODAS 2.0 has demonstrated excellent psychometric properties (29).

### Statistical Analyses

The statistical analyses were performed using IBM SPSS Statistics 26. The reliability was examined using Cronbach's alpha, mean inter-item correlation, and the test-retest correlation coefficient. The reliability analyses were calculated using the PG-13 symptom severity score.

Concurrent validity was assessed by examining associations between PG-13 and measures of PTSD (PCL-5 and CAPS-5), depression (MINI), and general psychological distress (SCL-27). Pearson's *r* was calculated for both concurrent validity and test-retest reliability.

The factor analyses included an exploratory and a confirmatory analysis. First, a principal component analysis (PCA) with oblique rotation was performed. The number of factors was determined by an evaluation of the scree plot and the eigenvalues. Confirmatory factor analyses were conducted using Mplus version 8 (30). Given the ordinal nature of the item response options, a robust weighted least-squares with a mean and variance adjustment (WLSMV) estimator was employed (31–33). Goodness of model fit was assessed with the χ^2^ test, the comparative fit index (CFI), the Tucker-Lewis Index (TLI), the root mean square error of approximation (RMSEA), and weighted root-mean-square residual (WRMR). The following standards were used in assessing model fit: a non-significant test of model χ^2^, χ^2^/df between 2 and 3, CFI and TLI >0.90, RMSEA <0.08 (34), and WRMR < 1.0 (35, 36).

*T*-tests were conducted to explore whether there was a difference in symptoms between individuals who reported bereavement as the traumatic index event and bereaved individuals who reported a traumatic index event not related to bereavement. To compare correlation coefficients, Fisher's *z*-tests were conducted. For participants missing one or two items on PG-13 (*n* = 3), the missing values were imputed using the estimated mean values on PG-13. The same procedure was used for missing data on SCL-27 (*n* = 15). Participants with more than 50% missing values on PG-13 were excluded from the study (*n* = 3).

## Results

### Sample Characteristics

In total, 123 participants were included in the study. A majority were women (80.5%) and the mean age was 37.85 years (SD = 14.97, range = 18–78). In all, 52.8% were working (*n* = 65), 23.6% (*n* = 29) were studying, and 65.8% (*n* = 81) had a high level of educational attainment (attended or finished university). Of those reporting that the loss was not the trauma index event (*n* = 51), 13 reported the event to be sexual violence, 12 reported somatic disease/injury, 10 reported accident, 10 individuals reported interpersonal violence, and 6 reported another traumatic event. The total sample mean score on PG-13 was 24.98 (SD = 8.48). Four participants reported no symptoms and only three participants screened positive for PGD. See Table 1 for descriptive statistics on all measures.

**Table 1:** Mean (SD) scores on symptoms of prolonged grief, posttraumatic stress, depression and general psychological distress, disability level, and comparison between the two subsamples.

			**Index event**		
		**All**	**Bereavement**	**Comparison**	**T-score^a^**
		**(*n* = 123)**	**(*n* = 72)**	**(*n* = 51)**	
PG-13		24.98 (8.47)	26.56 (8.35)	22.75 (8.23)	2.51^*^
PCL-5^b^		24.68 (17.46)	22.81 (16.01)	27.53 (19.30)	−1.42
CAPS-5		18.39 (12.28)	16.18 (10.34)	21.51 (14.11)	−2.30^*^
	Intrusion	4.76 (3.89)	3.93 (3.12)	5.92 (4.56)	−2.70^**^
	Avoidance	2.10 (2.02)	1.94 (1.93)	2.31 (2.14)	−1.00
	NACM	6.23 (4.80)	5.60 (4.24)	7.12 (5.42)	−1.74
	Hyperarousal	5.31 (3.54)	4.71 (3.17)	6.16 (3.88)	−2.20^*^
Depression^c^	Ongoing	1.42 (2.73)	1.27 (2.44)	1.66 (3.14)	−0.76
	Previous	5.34 (3.21)	5.03 (3.27)	5.81(3.09)	−1.30
SCL−27^b^		27.72 (21.40)	25.62 (21.35)	30.93 (21.48)	−1.30
	Depressive	5.16 (3.60)	4.87 (3.51)	5.60 (3.72)	−1.06
	Dysthymic	6.48 (4.56)	6.23 (4.85)	6.87 (4.10)	−0.73
	Somatization	5.64 (6.03)	5.13 (6.00)	6.00 (6.06)	−1.12
	Agoraphobic	3.32 (4.42)	2.65 (3.56)	4.36 (5.36)	−1.88
	Social phobic	3.76 (3.87)	3.68 (3.82)	3.89 (3.99)	−0.28
	Mistrust	3.63 (3.64)	3.25 (3.48)	4.22 (3.85)	−1.30
WHODAS 2.0^d^		15.98 (17.35)	13.15 (15.71)	20.23 (18.94)	−2.18^*^

*Means and standard deviation for all scales used in the study. Means presented for all participants, participants with bereavement as index event and participants with other index events (i.e., comparison group). PG-13, Prolonged Grief questionnaire-13; PCL-5, PTSD Checklist for DSM-5; CAPS-5, Clinician-Administered PTSD Scale for DMS-5; SCL-27, Symptom Checklist 27; WHODAS, World Health Organization Disability Assessment Scale*.

a *T-test of the difference of means between participants with bereavement as index event and participants with other index events*.

b *Missing data, n = 114 (69 and 45)*.

c *Missing data, n = 118 (71 and 47)*.

d *Missing data, n = 113 (69 and 44)*.

* *p < 0.05*,

** *p < 0.01*.

### Reliability

The internal consistency of the PG-13 was good, Cronbach's α = 0.86, and it was not improved by removal of any of the PG-13 items. The item-total correlations of the PG-13 items with the total score ranged from *r*_itc_ = 0.42 (item 9) to *r*_itc_ = 0.63 (item 11). The mean item-total correlation was *r*_itc_ = 0.55. The mean inter-item correlation was *r* = 0.36, range *r* = 0.12–0.75. The mean inter-item correlation was lowest between “*yearning*” (item 1) and “*trust in others*” (item 8), *r* = 0.12, and highest between the two *separation distress* items (items 1 and 2), *r* = 0.75.

A total of 84 participants completed and returned the retest questionnaire. The temporal stability of PG-13 was good, with high correlations for the total symptom score *r* = 0.86 (*p* < 0.001). The correlations ranged from *r* = 0.65 (*p* < 0.001) to *r* = 0.81 (*p* < 0.001) at the item level. The mean time for retest was 6.26 days (SD = 9.41 days, range = 0–50 days).

### Concurrent Validity

The correlations between PG-13 and the other measures of distress and function are shown in Table 2. In the total sample, there were low to moderate correlations between the PG-13 and the measure of related disorder. The strongest correlation was with the SCL-27 total score and the second strongest was with PCL-5.

**Table 2:** PG-13 correlations with symptoms of posttraumatic stress, depression, general psychological distress, and disability level, and comparison between the two subsamples.

			**Index event**		
		**All**	**Bereavement**	**Comparison**	***z*-value^a^**
		**(*n* = 123)**	**(*n* = 72)**	**(*n* = 51)**	
PCL-5^b^		0.52^***^	0.61^***^	0.42^**^	1.39^**^
CAPS-5		0.48^***^	0.79^***^	0.32^*^	3.94^***^
	Intrusion	0.33^***^	0.63^***^	0.20	2.87^**^
	Avoidance	0.49^***^	0.63^***^	0.39^**^	1.75^*^
	NACM	0.48^***^	0.73^***^	0.31^*^	3.24^**^
	Hyperarousal	0.38^***^	0.58^***^	0.29^*^	1.94^*^
Depression^c^	Ongoing	0.27^**^	0.44^**^	0.13	1.77^*^
	Previous	0.03	0.25^*^	−0.25	2.64^**^
SCL-27^b^		0.56^***^	0.71^***^	0.46^**^	1.98^*^
	Depressive	0.54^***^	0.65^***^	0.49^**^	1.21
	Dysthymic	0.52^***^	0.65^***^	0.38^*^	1.90^*^
	Somatization	0.42^***^	0.57^***^	0.29	1.77^*^
	Agoraphobic	0.46^***^	0.63^***^	0.45^**^	1.30
	Social	0.49^***^	0.60^***^	0.37^*^	1.54
	Mistrust	0.40^***^	0.56^***^	0.35^*^	1.36
WHODAS 2.0^b^		0.43^***^	0.62^***^	0.27	2.29^*^

*Correlations between PG-13 mean score and mean score on the other scales for all participants, participants with bereavement as index event and participants with other index events (i.e., comparison group). PG-13, Prolonged Grief questionnaire-13; PCL-5, PTSD Checklist for DSM-5; CAPS-5, Clinician-Administered PTSD Scale for DMS-5; SCL-27, Symptom Checklist 27; WHODAS, World Health Organization Disability Assessment Scale*.

a *Comparison of correlations (Z-test) between participants with bereavement and participants with other index events*.

b *Missing data, n = 114 (69 and 45)*.

c *Missing data, n = 113 (71 and 47)*.

d *Missing data, n = 115 (69 and 46)*.

* *p < 0.05*.

** *p < 0.01*.

*** *p < 0.001*.

### Construct Validity

#### Principal Component Analysis

The factor analysis rendered a significant Bartlett's test of sphericity (*p* < 0.001), the sampling adequacy was 0.83 (Kaiser-Meyer-Olkin), and 67.7% of the total variance was explained with three factors (Table 3). The first factor, labeled social/identity disruption, included four items and explained 42.9% of the variance. The second factor, labeled separation distress, included four items and explained 15.1% of the variance. The third factor, labeled traumatic distress, included three items and explained 9.8% of the variance (Table 3).

**Table 3:** Principal component analysis with oblique rotation of the PG-13.

**Items**	**Factor loadings**		
	**Factor 1: Reorientation/identity**	**Factor 2: Separation distress**	**Factor 3: Traumatic distress**
1. In the past month, how often have you felt yourself longing or yearning for the person you lost?		−0.85	
2. In the past month, how often have you had intense feelings of emotional pain, sorrow, or pangs of grief related to the lost relationship?		−0.89	
4. In the past month, how often have you tried to avoid reminders that the person you lost is gone?		−0.79	
5. In the past month, how often have you felt stunned, shocked, or dazed by your loss?		−0.73	
6. Do you feel confused about your role in life or feel like you don't know who you are?	0.83		
7. Have you had trouble accepting the loss?			0.52
8. Has it been hard for you to trust others since your loss?			0.64
9. Do you feel bitter over your loss?			0.87
10. Do you feel that moving on (e.g., making new friends, pursuing new interests) would be difficult for you now?	0.75		
11. Do you feel emotionally numb since your loss?	0.74		
12. Do you feel that life is unfulfilling, empty, or meaningless since your loss?	0.78		
Eigenvalues	4.7	1.7	1.1
Explained variance (%)	42.9	15.1	9.8

#### Confirmatory Factor Analysis

Based on the principal content analysis suggesting three factors, the theoretically derived two-factor model (37) and previous research supporting a one-facture model for prolonged grief (2, 9, 10, 18–20), three models with 1, 2, and 3 factors, respectively, were tested. The two- and three-factor models yielded acceptable model fit; however, the three-factor model performed slightly better (Table 4 presents model fit indices).

**Table 4:** Fit indices for the confirmatory factor analyses of the PG-13.

**Model**	**χ^2^**	**df**	**CFI**	**TLI**	**RMSEA [90% CI]**	**WRMR**
One factor	193.60^***^	44	0.89	0.87	0.17 [0.14; 0.19]	1.32
Two factors	81.69^***^	43	0.97	0.97	0.09 [0.06; 0.11]	0.75
Three factors	69.62^**^	41	0.98	0.97	0.08 [0.04; 0.11]	0.66

** *p < 0.01*.

*** *p < 0.001*.

*Df, degrees of freedom; CFI, Comparative fit index; TLI, Tucker-Lewis Index; RMSEA, Root mean square error of approximation; WRMR, Weighted root-mean-square residual*.

### Between-Group Comparison

The bereavement group had significantly higher scores on PG-13 than the comparison group (Table 1). The total scores for PCL-5, CAPS-5, and WHODAS were significantly higher for the comparison group than for the bereavement group, whereas there were no significant differences between groups on SCL-27 or depression (Table 1). As noted in Table 2, the correlations in the bereavement group were stronger than in the comparison group for the majority of the measures, excepting only some of the subscales of SCL-27. In the comparison group, the correlations were non-significant between PG-13 and the PCL-5 intrusion subscale and the SCL-27 somatization subscale. This was also the case for both the interview-based depression score and the functional disability score. In contrast, in the bereavement group, all correlations for PG-13 were significant, except that with previous depression.

## Discussion

This study examined the psychometric properties of the PG-13 and the latent structure of prolonged grief in a Swedish sample. The Swedish PG-13 possessed adequate internal consistency and mean inter-item correlations as well as temporal stability at both the scale and item levels. Support of concurrent validity of PG-13 was shown by significant positive correlations with measures of posttraumatic stress, depression, and general psychological distress. This is consistent with there being overlap in symptoms between prolonged grief, depression, and posttraumatic stress, which are known to correlate to some extent (18, 38). Nevertheless, PGD, depression, and PTSD are distinct clinical entities (21).

The factor analyses provided stronger support for models with two or three factors, as compared to a unidimensional model of prolonged grief. The two-factor model yielded slightly poorer fit than the three-factor model. The two-factor model encompassed a separation distress factor, including yearning, which is one of the core symptoms of prolonged grief, and a second factor including items that could be understood as manifestations of traumatic distress and items of social/identity disruption, such as confusion about finding a new role in life, difficulty to move on, and feeling that life is empty after the loss. In the three-factor model, the factors were social/identity disruption, separation distress, and traumatic distress. These findings may reflect the sample composition and they diverge from previous studies supporting a unidimensional structure of PG-13 (2, 18) and studies on other measures of prolonged grief examining the underlying structure of prolonged grief (2, 9, 10, 19, 20). However, the study by O'Connor et al. (37) found support for the theoretically derived two-factor model, in agreement with the present study. A recent study on a Hebrew version of Inventory of Complicated Grief among older bereaved parents found support for a three-factor structure (39). It should be noted that the fit of the three-factor model in the present study may be positively biased because the fit was assessed on the same sample that was used in the exploratory factor analysis. The possible positive bias for the fit of the three-factor model and the small differences in model fit between the two-factor and three-factor models speak in favor of the two-factor model.

To the authors' knowledge, this is one of few studies examining differences in symptom severity of PTSD and other psychiatric illnesses between bereaved individuals with and without loss as the primary index event. Individuals who reported bereavement as their index event had higher levels of grief, and lower symptoms of PTSD and functional disability as compared to individuals that reported a different event than the loss as an index-event. In addition, there were overall lower associations between grief and the other measures in the comparison group. This suggested that prolonged grief symptoms were more strongly associated with symptoms of PTSD and depression when they arose from the same event. It also suggested that PTSD symptoms were slightly more pronounced and functional disability was markedly higher if the traumatic index event was not the same as the precipitating event for the PGD symptoms. Although these findings are tentative, they provide an interesting picture of the dynamics between prolonged grief symptoms and other forms of distress and functional disability related to the type of traumatic index event.

Some limitations of this study warrant mention. Low overall symptom scores may have caused inflated correlations. Only three participants screened positive for PGD, a rate that is lower than in previous studies (7, 8, 22, 40). Additionally, the TRACES study was not primarily aimed toward bereavement and thus no additional questions were asked about the nature of the relationship between the participant and the deceased other than if it was a significant other. Another limitation is that convergent validity was not investigated. The small sample size limits the generalizability of the findings, which need to be replicated in larger samples with higher overall symptom burden. However, the findings are likely reflective of the wide range of reactions that may arise after experiencing loss, with the majority of those affected recovering well and a minority developing long-term distress. Finally, although a factor model should be validated through exploratory analysis on an independent sample, the sample size precluded such independent validation in this study. The model fit of the three-factor model should be viewed in light of this. This does not impact the advantage of the two-factor model over the unidimensional model.

In conclusion, the study supports the use of the Swedish PG-13 to measure prolonged grief in bereaved adults. The Swedish PG-13 was shown to have good reliability, including temporal stability, and findings supported construct and concurrent validity. The latent structure of prolonged grief suggested two or three factors, and not a unidimensional structure, which has been suggested previously. Overall, the PG-13 is a useful instrument in research, to increase the understanding of prolonged grief in adults. Although, due to the limitations of the study, including the small sample size and not assessing convergent validity the results should be interpreted with caution. Future research is needed in clinical samples to confirm the evidence for validity of the Swedish PG-13.

## Data Availability Statement

The datasets generated for this study are available on request to the corresponding author.

## Ethics Statement

The studies involving human participants were reviewed and approved by Uppsala Regional Ethics committee. The patients/participants provided their written informed consent to participate in this study.

## Author Contributions

JS, KB, FA, and JH contributed to conception and design of the study. KB organized the database. JS performed the statistical analyses. JH and JS wrote the first draft of the manuscript. KB and FA wrote sections of the manuscript. All authors contributed to manuscript revision and read and approved the submitted version.

## Conflict of Interest

The authors declare that the research was conducted in the absence of any commercial or financial relationships that could be construed as a potential conflict of interest.
